# A multicentre, prospective, randomized, open-label pragmatic trial to compare the effectiveness and safety of interferon beta-1a and glatiramer-acetate in paediatric patients affected by Multiple Sclerosis

**DOI:** 10.1007/s10072-025-08377-3

**Published:** 2025-08-08

**Authors:** Marta Simone, Roberto Palumbi, Mariaclara Achille, Stefania Micella, Mara Pascali, Antonia Peschechera, Alessandra Gabellone, Lucia Marzulli, Andrea De Giacomo, Giulia Maggi, Giorgio Reggiardo, Mariagrazia Felisi, Rosa Padula, Donato Bonifazi, Massimiliano Valeriani, Laura Papetti, Roberta Lanzillo, Valentina Torri Clerici, Lucia Margari

**Affiliations:** 1 Child Neuropsychiatry Unit, Department of Precision and Regenerative Medicine and Jonic Area (Dimepre-J), University of Bari “Aldo Moro, Bari, Italy; 2 Child Neuropsychiatry Unit, Department of Translational Biomedicines and Neurosciences (DiBrain), University of Bari “Aldo Moro, Bari, Italy; 3 Consorzio Per Valutazioni Biologiche e Farmacologiche (CVBF), Bari, Italy; 4 TEDDY, European Network of Excellence for Paediatric Research, Pavia, Italy; 5 Developmental Neurology Unit, Bambino Gesù Children’s Hospital, IRCCS, 00165 Rome, Italy; 6 Systems Medicine Department, University of Tor Vergata, Rome, Italy; 7 Translational Pain Neuroscience and Precision Medicine, Department of Health Science and Technology, School of Medicine, CNAP, Aalborg University, Aalborg, Denmark; 8 Department of Neurosciences and Reproductive and Odontostomatological Sciences, University of Naples Federico II, Naples, Italy; 9 Foundation Neurological Institute C. Besta, Milan, Italy

**Keywords:** POMS, Interferon beta-1a, Glatiramer acetate, Phase III randomized clinical trial, Pragmatic study

## Abstract

**Background:**

Paediatric-onset Multiple Sclerosis (POMS) is a rare, highly active disease requiring timely disease-modifying therapy.

**Objectives:**

This trial compared the efficacy and safety of intramuscular (i.m.) Interferon beta-1a (IFN-beta 1a) and subcutaneous (s.c.) Glatiramer Acetate (GA).

**Methods:**

A 96-week, Phase IIIb, multicenter, open-label randomized trial enrolled 30 participants (ages 12–17). Patients were randomized into two groups: 15 received i.m. IFN-beta 1a, and 15 received s.c. GA. The primary endpoint was MRI disease activity-free status; secondary outcomes included annualized relapse rate (ARR), time to first relapse, Expanded Disability Status Scale (EDSS), cognitive and fatigue scores, and quality of life.

**Results:**

The groups differed by age but not sex. MRI disease activity-free status did not differ (*p* = 0.09). ARR was significantly lower in the GA group (0.20; 95% CI 0.08–0.42) than in the IFN-beta 1a group (0.57; 95% CI 0.31–0.95, *p *= 0.02). EDSS, cognitive, fatigue, and quality-of-life scores were comparable. Notably, 50% of GA patients and 30% of IFN-beta 1a patients switched to higher efficacy treatments.

**Conclusions:**

Both treatments showed similar efficacy and safety, but early high-efficacy therapy may be preferable for POMS management.

**Trial Registration:**

EudraCT Number: 2017–005129-18. https://www.clinicaltrialsregister.eu/ctr-search/trial/2017-005129-18/IT

## Introduction

Paediatric-onset multiple sclerosis (POMS) is a rare condition, with an estimated prevalence between 0.13 and 0.6/100.000 children [1, 2]. Approximately 3% to 5% of all cases of MS affect paediatric patients, most of whom are teenagers aged 13 and over [3, 4]. Children and adolescents with POMS have an active inflammatory disease course with a higher relapse rate, higher lesion accrual at magnetic resonance imaging (MRI), and more prominent cognitive impairment (CI) than adult-onset MS (AOMS) [5]. The early onset of the disease, before age 18, alters brain development and significantly impacts patients'quality of life and academic or professional goals [6].

Moreover, POMS patients reach significant disability milestones, such as an Expanded Disability Status Scale (EDSS) score of 6.0, at a younger age in comparison to AOMS, suggesting that POMS should not be regarded as a more benign disease in comparison to its adult counterpart [7–9].

This backdrop highlights the importance of timely intervention with disease-modifying therapy (DMT). Because of low disease prevalence and difficulties in patient recruitment into randomized clinical trials (RCTs), obtaining long-term safety and efficacy data for regulatory approval of DMTs is challenging in POMS. As a result, DMTs licensed for use in adults, including interferons beta (IFN beta 1a), dimethyl fumarate (DMF), fingolimod, glatiramer acetate (GA), natalizumab, and teriflunomide, have been used off-label up in POMS until recent years. This trend is changing, as licensed therapies for paediatric patients are now emerging following the positive results from RCTs: Fingolimod was approved in 2018 by the US Food and Drug Administration and the European Medicines Agency for patients with MS aged 10 to 17 years, Teriflunomide was approved by the European Medicines Agency in 2021 for use in patients with relapsing–remitting MS (RRMS) aged 10 to 17 years, and dimethyl fumarate was approved in 2022. The 2024 Operetta 2 trial, currently ongoing, aims to approve Ocrelizumab in patients with POMS [10–12]. However, until 2018, IFN beta 1a and GA were the only treatments approved for patients with POMS older than 12 years, since their positive effect in reducing relapse rate and delaying disability progression, with a generally favourable safety profile, was demonstrated in retrospective and prospective observational studies [13–15]. A comparison of the ability to control disease activity and disability accrual between these two drugs has never been assessed in POMS in real life. Therefore, in 2018, we started a Phase IIIb, 24-month multicentre, pragmatic, open-label, randomized trial aimed at evaluating the equivalence or the superiority of weekly administered intramuscular (i.m.) IFN beta1a 30 mcg and daily administered subcutaneously (s.c.) GA 20 mg in reducing clinical and MRI activity in POMS. We chose this study design because it offers several advantages. Firstly, the parameters analyzed are tailored to real-world experience, hence exploring measures that can potentially enhance treatment adherence. Therefore, the results may be easily translated into the clinical practice, and this feature is very useful for trials involving a rare disease as POMS. Lastly, pragmatic studies examine patient-relevant outcomes as these are pivotal to decision-making, rather than focusing on more didactic outcomes.

## Methods

### Trial design

This study is a Phase IIIb, 24-month multicentre, pragmatic, prospective, open-label, randomized trial aimed at demonstrating the equivalence or superiority of IFN beta-1a 30 mcg weekly i.m. and GA 20 mg daily s.c., in relapsing–remitting (RR) POMS (The trial was registered with www.clinicaltrialsregister.eu, EudraCT Number: 2017–005129-18). We used the CONSORT reporting guidelines for randomized trials [16].

Nineteen recruiting centers were invited to participate in this trial. Among them, four centers were able to recruit patients: the University Hospital of Bari (Italy), Bambino Gesù Children Hospital of Rome (Italy), the University of Naples Federico II (Italy), and the Foundation Neurological Institute C. Besta (Milan, Italy).

Treatment naïve RR POMS with at least one MS relapse during the previous year, and an EDSS score at onset between 0 and 5.5 were included. Patients with progressive MS, autoimmune comorbidities, or other medical conditions (such as acute disseminated encephalomyelitis mood disorders, thyroid dysfunctions, and severe renal insufficiency) were excluded.

Patients were randomized to one of the two treatment arms (arm A: IFN beta-1a; arm B: GA), in a 1:1 ratio, using a randomization list produced by a study statistician using SAS PROC PLAN [17, 18]. Randomization was performed on the same day of pre-dose determination; outcomes were routinely recorded in the Italian Multiple Sclerosis Registry [19] and subsequently in an eCRF.

The interventional study included two phases: a pre-randomisation screening phase, to collect informed consent and confirm patients’ study eligibility, and a treatment phase starting on Day 1 with randomization to one of the two treatment arms. Investigational medical products were administered according to the randomization arm, within 15 days of the screening procedures. Site personnel provided training to the patients and their parents/caregivers on the correct procedure for the administration of i.m. and s.c. injections.

The primary outcome was the proportion of patients free of new or newly enlarging T2 hyperintense on brain MRI scans at 96 weeks.

Secondary outcomes included the annualized Relapse Rate (ARR) at Week 96, the proportion of subjects free of relapse up to Week 96, time to First Relapse and changes of baseline EDSS, Fatigue Severity scale (FSS), Symbol Digit Modality Test (SDMT), paediatric quality of life (ped QOL) scores at Week 96.

### Visit schedule

Post-randomization visits were scheduled at 2, 24, 48, 72 and 96 weeks**.** Patients were treated for 96 weeks. Clinical evaluation was performed at baseline and every six months and included: complete medical history, physical and neurological examination, with disability assessed by EDSS, ARR (including, at baseline, the ARR in the year prior randomization), quality of life measured by the PEDQoL scale, fatigue assessed by the FSS, vital signs, hematological/blood chemistry, the occurrence of severe adverse events (SAEs) and Adverse Events (AEs), urinalysis and pregnancy test (beta hCG).

Brain MRI scan (by 1.5 T MRI), and cognitive function assessment, using the SDMT were assessed at baseline and every 12 months.

### Statistical analysis

Descriptive statistics were used for demographic and clinical variables. Continuous variables were summarized by treatment arm (GA vs. IFN beta 1a) using mean, standard deviation, median, minimum, and maximum, while categorical variables were presented as frequency distributions with 95% confidence intervals when appropriate.

The primary endpoint, the proportion of patients free of new/enlarging T2 hyperintense lesions at 96 weeks, was assessed by a logistic regression (LR) analysis.

ARR at Week 96 was calculated using the person-years method and analyzed by a negative binomial regression, adjusting for baseline relapse rate, EDSS, and age. The Kaplan–Meier survival method was proposed to analyze the time to the first relapse.

Changes in FSS, EDSS, SDMT, and ped QOL scores from baseline to Week 96 were analyzed by General Linear Models (GLM). Adverse events (AEs) and adverse drug reactions (ADRs) were descriptively analyzed and coded using MedDRA. No imputation was performed for missing data. A finalized Statistical Analysis Plan (SAP) outlined methods, endpoints, and reporting formats, with protocol deviations documented in the Clinical Study Report.

## Results

**Fig. 1:**
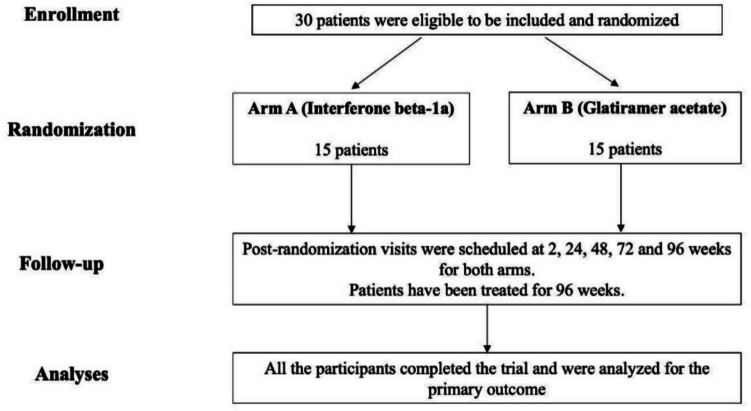
Participant flow diagram

The study cohort included 30 treatment naïve RR POMS who were randomized to one of the two treatment arms in a 1:1 ratio (Fig. 1). Fifteen patients were randomized to weekly i.m. IFNbeta 1a (mean age = 14.7 + 1.50; 73% women) and 15 patients to daily s.c. GA (mean age = 15.8 + 1.05; 71% women). The baseline demographic and clinical characteristics of the study population by treatment group are presented in Table 1.

**Table 1:** Baseline demographic and clinical characteristics of study population by treatment group

	Statistic	GA (*N* = 15)	IFN-beta1a (*N* = 15)	*p-*value
Age at screening (years)	Mean (SD)	15.8 (1.05)	14.7 (1.50)	0.330*
	Median	16.0	15.0	
	Min, Max	13, 17	12, 17	
Age at onset (years)	Mean (SD)	15.71 (1.07)	14.40 (1.45)	0.100*
	Median	16.0	14.0	
	Min, Max	13, 17	12, 16	
Duration of disease (years)	Mean (SD)	1.71 (2.13)	2.87 (2.64)	0.700*
	Median	1.0	2.0	
	Min, Max	0, 8	0, 8	
Sex				
Female	n (%)	10 (71.4^1^)	11 (73.3^1^)	0.592**
Male	n (%)	4 (28.6^1^)	4 (26.7^1^)	
Unknown	n (%)	1	0	
MRI at baseline				
Yes	n (%)	14 (100%)	15 (100%)	-
No	n (%)	0 (0%)	0 (0%)	
Unknown	n (%)	1	0	
Number of T2 hyperintense lesions at baseline	Mean (SD)	11.38 (9.2)	10.93 (9.2)	0.706*
	Median	7.0	6.0	
	Min, Max	4, 30	1, 30	
Number of T1 Gd-enhancing lesions at baseline	Mean (SD)	0.57 (1.2)	2.20 (4.2)	0.450*
	Median	0.0	0.0	
	Min, Max	0, 4	0, 15	
EDSS at baseline	Mean (SD)	1.75 (0.67)	1.77 (0.86)	1.000*
	Median	2.0	2.0	
	Min, Max	0.0, 2.5	0.0, 3.0	

^1^ Percentage is calculated on patients with available data

* For the calculation of this *p*-value a non-parametric test (Wilcoxon) has been applied

** For the calculation of this *p*-value a Pearson’s Chi-square test has been applied

### Primary endpoint analysis

**Fig. 2:**
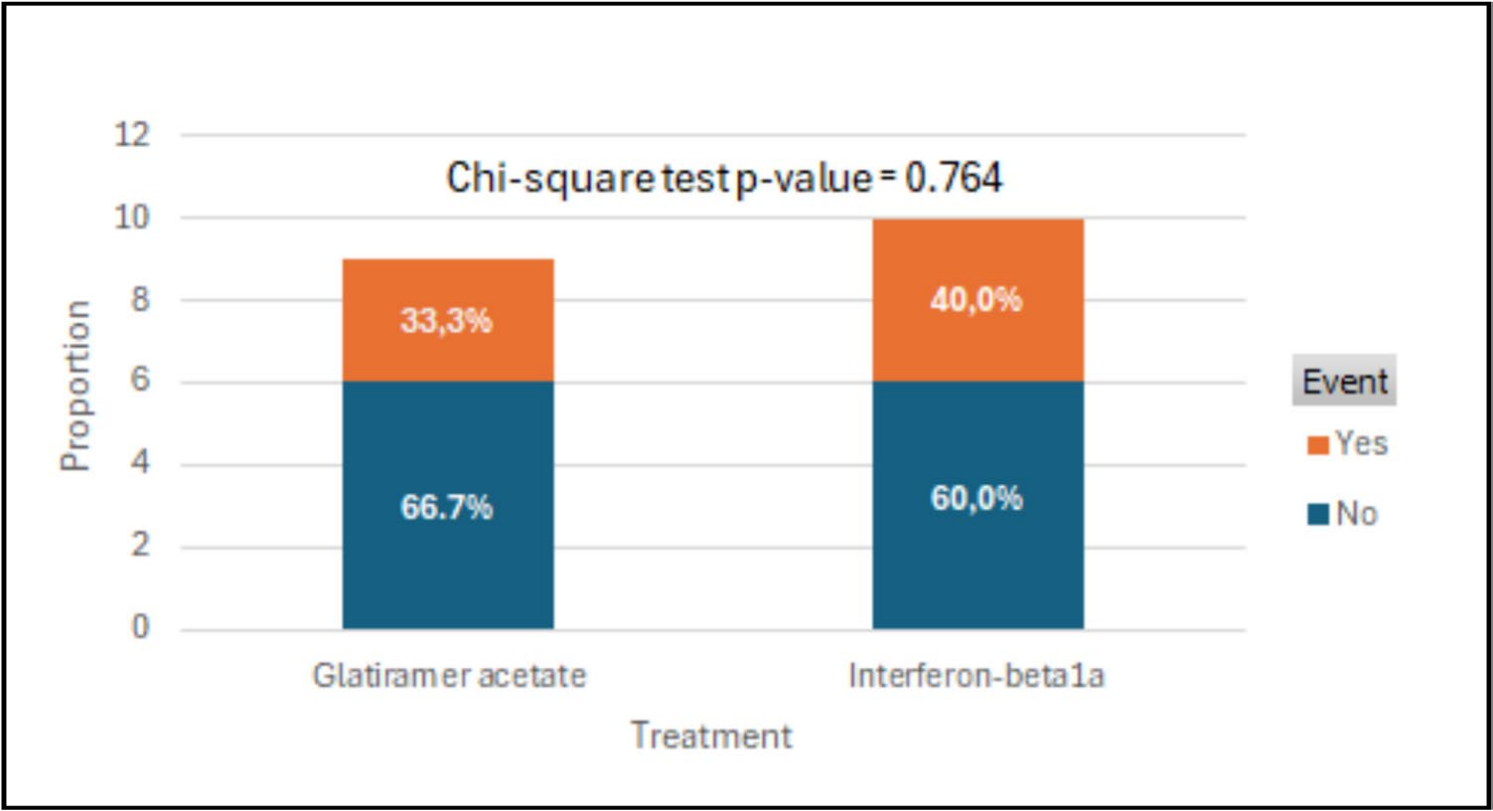
Proportion of patients free of new or newly enlarging T2 hyperintense on brain MRI scans at 96 weeks

The percentage of patients free from new or newly enlarging T2 hyperintense on brain MRI scans at 96 weeks (Fig. 2) did not differ between the two treatment groups (33.3% in the GA group and 40% in IFN beta 1a group; chi-framework test, *p*-value = 0.09).

As shown in Table 2, the Odds Ratio for treatment is 1.00 (95% CI: 0.395, 2.534), indicating that the odds of event for patients treated with GA are the same as the patients treated with IFN beta1a.

**Table 2:** Logistic Regression analysis results

Variables in the Equation								
	B	S.E	Wald	df	Sig	Exp(B)	95% C.I. for EXP(B)	
							Lower	Upper
Treatment (GA)	0.000	0.949	0.000	1	1.000	1.000	0.395	2.534
Constant	0.223	1.500	0.022	1	0.882	1.250	-	-

### Secondary endpoint analysis

Comparison of total number of relapses, total number of person-years at risk, median ARR, and median ARR difference between the two treatment groups are reported in Table 3.

**Table 3:** Comparison of Total number of relapses, Total number of person-years at risk, Median ARR, and Median ARR Rate difference between the two treatment groups

Treatment	Total number of relapses	Total number of person-years at risk	ARR median (95% C.I.)	Median ARR Rate difference (95% C.I.)	*p*-value
GA	7	34.97	0.2001 (0.0827,0.4241)	−0.3698 (−0.6762, −0.0633)	**0.018**
Interferon beta-1a	14	24.56	0.5699 (0.3116,0.9564)		

ARR was significantly lower (*p* = 0.018) in the GA group (0.2001; 95% CI 0.0827–0.4241) than in the IFN beta 1a group (0.5699; 95% CI 0.3116, 0.9564) at week 96. The median ARR Rate difference was −0.3698 (95% C.I. −0.6762, −0.0633).

**Fig. 3:**
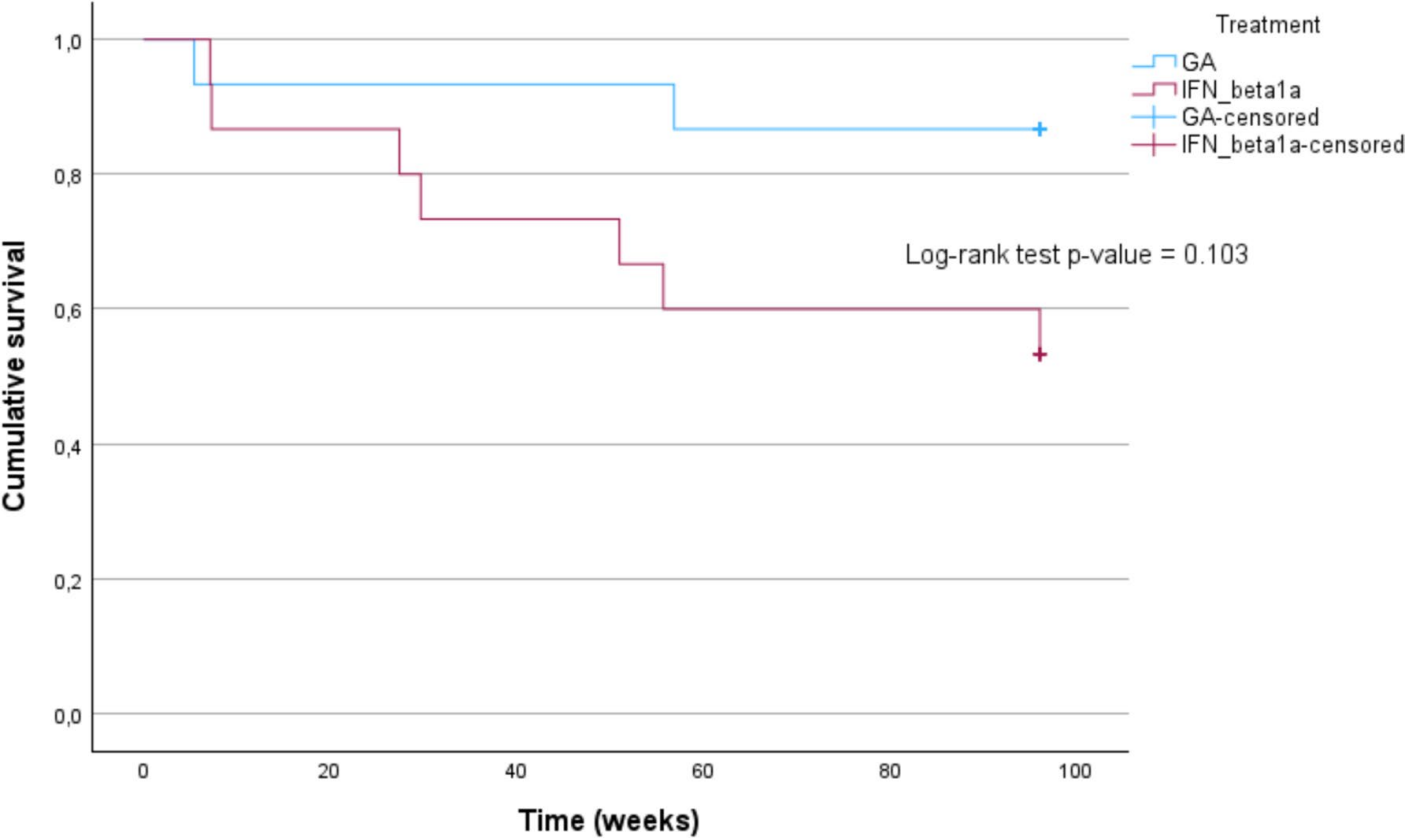
Time to first relapse, assessed through Kaplan–Meier curves

Time to first relapse, assessed through Kaplan–Meier analysis (Fig. 3), trended (Log-rank test; *p* = 0.103) to be longer in GA in comparison to the IFN beta 1a group., but the difference did not reach statistical significance.

Similarly, the GLMs showed that score variations between basal and visit 5 for FSS (*p* = 0.555), EDSS (*p* = 0.661), SDMT, *p* = 0.375) and the PEDQoL (*p* = 0.662) were not different between the two treatment groups, as shown in Tables 4.

**Table 4:** GLM Analysis: FSS, EDSS, SDMT, PedQoL difference from baseline (Visit 5 vs Baseline)

Measure	Treatment Group	Baseline (Week 0) Mean (SD)	Baseline (Week 0) Median (Min, Max)	Visit 5 (Week 96) Mean (SD)	Visit 5 (Week 96) Median (Min, Max)	Difference from Baseline Mean (SD)	Difference from Baseline Median (Min, Max)	*p*-value
Fatigue Severity Scale (FSS)	**Glatiramer Acetate**	23.92 (7.89)	24.00 (12, 35)	26.75 (15.84)	29.00 (9, 46)	4.50 (10.47)	7.50 (−13, 14)	0.555
	**Interferon beta-1a**	17.42 (6.47)	18.00 (9, 29)	27.29 (18.27)	23 (9, 54)	6.17 (13.24)	5.50 (−13, 25)	
	**Total**	20.67 (7.80)	19.00 (9,35)	27.00 (16.37)	27.00 (9, 45)	5.21 (11.27)	5.50 (−13, 25)	
Expanded Disability Status Scale (EDSS)	**Glatiramer Acetate**	1.75 (0.67)	2.00 (0.0, 2.5)	1.79 (0.27)	2.00 (1.5, 2.0)	−0.14 (0.47)	0.00 (−1.0, 0.5)	0.666
	**Interferon beta-1a**	1.77 (0.86)	2.00 (0.0, 3.0)	1.69 (0.92)	1.75 (0.0, 3.0)	−0.25 (0.46)	−0.25 (−1.0, 0.5)	
	**Total**	1.76 (0.76)	2.00 (0.0, 3.0)	1.73 (0.68)	2.00 (0.0, 3.0)	−0.20 (0.45)	0.00 (−1.0, 0.5)	
Symbol Digit Modality Test (SDMT)	**Glatiramer Acetate**	59.29 (21.79)	55.00 (28, 100)	51.63 (16.03)	49.00 (35, 81)	0.00 (7.21)	−0.50 (−9, 9)	0.375
	**Interferon beta-1a**	50.50 (23.94)	46.50 (18, 120)	47.38 (15.92)	50.00 (18, 66)	3.71 (5.85)	1.00 (0, 16)	
	**Total**	54.89 (22.90)	53.50 (18, 120)	49.50 (15.59)	49.00 (18, 81)	1.73 (6.66)	1.00 (−9, 16)	
Pediatric Quality of Life (PedQoL)	**Glatiramer Acetate**	17.08 (8.72)	17.00 (1, 34)	19.00 (14.45)	23.50 (1, 43)	4.63 (12.03)	4.00 (−9, 24)	0.662
	**Interferon beta-1a**	18.00 (10.76)	16.50 (0, 34)	20.71 (15.91)	13.00 (5,48)	1.50 (13.00)	−5.00 (−8, 23)	
	**Total**	17.54 (9.59)	17.00 (0, 34)	19.80 (14.62)	23.00 (1, 48)	3.29 (12.06)	−1.00 (−9, 24)	

### Safety evaluation

Only a descriptive analysis on safety evaluation has been performed, as shown in Table 5. No SAEs occurred. No unexpected AEs were observed. The incidence of AEs was 20% (3/15) in the IFN beta group and 13% (2/15) in the GA group. All adverse events were mild and resolved within a few days. Only 1 AE (drug hypersensitivity reaction) in the GA group led to withdrawal of the drug. The overall safety of the two drugs was satisfactory. No other significant adverse events occurred.

**Table 5:** Safety evaluation

Adverse Events (Prospective cohort)								
ID	Arm	AE	Duration (Days)	Relationship to study drug	Severity	Study drug action	Other action taken	Outcome
2–1	Interferon beta-1a	Headache	1	No reasonable possibility of a relatedness with study medication	Mild	Dose not changed	None	Recovered/Resolved
2–1	Interferon beta-1a	Headache	1	No reasonable possibility of a relatedness with study medication	Mild	Dose not changed	New medication/non-drug therapy added	Recovered/Resolved
2–1	Interferon beta-1a	Fever	1	No reasonable possibility of a relatedness with study medication	Mild	Dose not changed	New medication/non-drug therapy added	Recovered/Resolved
2–5	Glatiramer Acetate	Drug hypersensitivity reaction	9	Reasonable possibility of a relatedness with study medication	Mild	Drug withdrawn	Concomitant medication/non-drug therapy changed or discontinued	Recovered/Resolved
2–6	Glatiramer Acetate	Tingling paresthesias	1	No reasonable possibility of a relatedness with study medication	Mild	Dose not changed	None	Recovered/Resolved

In addition, it has been observed that 46.7% of patients under GA and 33.3% under INFb-1a switched to higher efficacy treatments during the follow-up in a median time of 14.25 months (Min 4 months, max 24 months). In the GA group, 2 patients switched to Fingolimod, 2 to Teriflunomide, 1 to Ocrelizumab, 1 to Natalizumab, 1 to IFN beta 1b; in the IFB beta1a group, 1 switched to Fingolimod, 2 to Teriflunomide, and 2 to Ofatumumab.

**Fig. 4:**
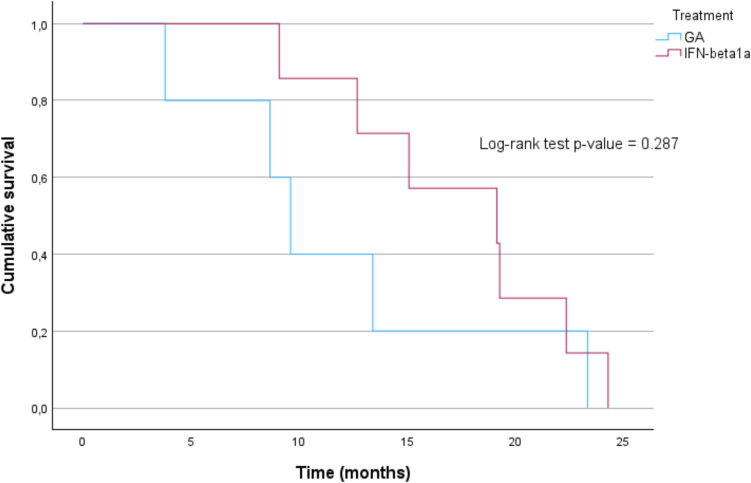
Cumulative survival function (event = treatment switch)

Furthermore, the difference in time to treatment switch was not statistically significant between the two groups (median = 9.6 months for IFN beta, 19 months for GA.), as estimated by applying the Kaplan–Meier (Fig. 4).

## Discussion

In this prospective, pragmatic, multicentre, randomized study we demonstrated a substantial equivalence of effectiveness between weekly i.m. IFN beta 1a and daily s.c. GA in POMS. Indeed, our study showed that both drugs had a similar effect in controlling MRI and clinical disease activity and physical and cognitive disability accrual, with a generally favourable safety and patient-reported outcome profile.

The only feature that differed between the two treatment groups was the ARR, which was lower in the GA cohort than in the IFN beta 1a group at the end of the 2-year follow-up. Similar results were found in a previous large head-to-head real-world observational study [20] in AOMS, comparing the effectiveness of IFN beta 1a and GA on clinical disease activity outcomes. This study showed a slightly lower relapse rate among patients treated with GA, but no discernible differences in disability outcomes at the end of the 12-month follow-up period were found. The novelty of this paper lies in the use of a pragmatic trial, which is an RCT embedded into usual care, so it has the advantage of being conducted in real-world settings while retaining the strengths of the experimental approach, including randomization [21].

Pragmatic trials, indeed, are designed to measure the degree of beneficial effect under “real world” clinical settings, considering also health-related, patient-relevant outcomes. They allow to directly inform treatment decisions in practice and aim to answer the question of what the best treatment choice is, conversely to traditional trials that investigate the interventions themselves. This is particularly relevant in POMS, as this condition has peculiar features that can affect traditional trial results: first, its rarity, which poses an intrinsic limitation, given the small numerosity of cohorts; secondly, the challenges that paediatric age poses, such as the need for a multidisciplinary approach or the involvement of the family, all of which can impact protocol adherence.

Unfortunately, so far, only few and mostly small pragmatic trials exist in MS because of its challenging application in clinical practice [22] and a small number of clinical trials have been completed in POMS. Until 2018, IFN beta 1a and GA were the only treatments approved in POMS [13–15, 27]. Thereafter Fingolimod in 2018, Teriflunomide in 2021, and dimethyl-fumarate in 2022 were approved, following positive RCTs results, for use in patients aged 10 to 17 years. The 2024 Operetta 2 trial, currently ongoing, aims to approve Ocrelizumab in patients with POMS [10–12].

Clinical trials are particularly challenging in the pediatric population, as shown by a position paper by IPMSSG [28] and in a recent commentary on the state of the art in POMS treatment [29]. Many factors can contribute to this intrinsic difficulty: first, the scarce numerosity of patients affected, given how POMS is a rare disease, especially compared to its adult counterpart.

This impacts the recruitment numbers, making these studies sometimes at risk of becoming “shadow studies”, with few patients enrolled, and thus not particularly cost effective, given how pediatric patients often require a multidisciplinary team (e.g. neurologist, pediatrician).

Moreover, the need of family involvement: although children appear to be prone to contributing, parents are often skeptical, citing fear of receiving a less efficient or a newer, less studied treatment as a reason to hesitate to participate in RCTs. At the same time, practical, real life elements such as work and school leave, can interfere with study schedules.

Lastly, safety plays a relevant role, being the most relevant feature to observe in children: this implies the need for longer, more prolonged open label studies or registries, delaying even more the timeline of RCTs and, consequently, of study results in terms of drug efficiency.

It is worth noting that our findings demonstrated a significant percentage of patients switched to high-efficacy treatments (HETs) during the observation period. Indeed, 30–50% of patients needed to switch to higher efficacy therapies due to partial control of the disease activity (occurrence of relapse or new or newly enlarging T2 hyperintense on brain MRI) suggesting that an early administration of HET may be a more favourable approach to the treatment of POMS, as in adult-onset MS. This further exemplifies how POMS is far from a benign condition. Indeed, not only an early DMT initiation is recommended in POMS [22], but also an early start of HET to achieve the greatest benefit in reducing the risk of disability worsening has been demonstrated in recent studies [23–25].

However, controversies persist regarding the pursuit of such an aggressive strategy due to potential longer-term side effects. This issue is particularly relevant in paediatric patients, as they are exposed to medications during key periods of growth and body development [26]. Therefore, it is recommended that POMS treatment should be carefully monitored for clinical response and safety evaluation.

The major limitation of this study lies in the small number of patients included and the lack of long-term effectiveness and safety data. The lack of statistical significance in the outcome differences could be related to the small sample size. Among the future perspectives, there is the intention to combine the results of this prospective study with retrospective data coming from the Italian Multiple Sclerosis Registry to compare and strengthen the results.

The results of this study provide information for therapeutic decisions in clinical practice for POMS. Although on a small sample size, this study demonstrated a substantial equivalence of efficacy between a weekly administered drug and a daily administered drug in POMS. Proving this equivalence may be particularly significant, as the former may be better accepted by young patients, increasing adherence to therapy.

Moreover, the innovative pragmatic randomized study design applied in this trial should be further applied to compare more recently approved oral DMTs (Fingolimod, Teriflunomide, and Dimethyl-fumarate) with early HETs (anti-CD20 monoclonal antibodies, Natalizumab, and cladribine) for POMS to reach the highest possible grade of personalized treatment: recent literature, both from observational studies performed on registry data and from RCTs, underlines that, despite both METs and HETs are effective and well tolerated in ped-MS patients, HETs appear to be significantly more efficacious, with an overall reassuring safety profile [30]. The choice of the most appropriate therapy for the patient is challenging for the physician and crucial for the patient, but especially in paediatrics it is often guided only by the physician's personal experience, indirect comparative data, drug availability, and cost concerns.

## Data Availability

After a reasonable request, access to the safety data of this study might be granted by the authors.
